# Learning radiotherapy: the state of the art

**DOI:** 10.1186/s12909-020-02054-z

**Published:** 2020-05-11

**Authors:** Gerard M. Walls, Gerard G. Hanna, James J. McAleer

**Affiliations:** 1 Cancer Centre Belfast City Hospital, Belfast Health & Social Care Trust, Belfast, Northern Ireland; 2 Patrick G Johnston Centre for Cancer Research, Queen’s University Belfast, Belfast, Northern Ireland; 3 Sir Peter MacCallum Department of Oncology, University of Melbourne, Melbourne, Australia; 4 Centre for Medical Education, Queen’s University Belfast, Belfast, Northern Ireland

**Keywords:** Clinical oncology, Radiation oncology, Medical education, Training, Apprenticeship

## Abstract

**Background:**

The last two decades have seen revolutionary developments in both radiotherapy technology and postgraduate medical training. Trainees are expected to attain competencies using a mix of experiential learning, formal postgraduate teaching, self-directed learning and peer education. Radiation (Clinical) Oncology is a recognised ‘craft specialty’ where the apprenticeship model of training is applicable. This scoping review examines the evidence in relation to how medical trainees learn radiotherapy.

**Methods:**

A systematic search of MEDINE and EMBASE was undertaken to identify studies of trainee and/or trainer experience of radiotherapy learning published 1999–2018. Results pertaining to Medical Oncology, workforce trends, undergraduate radiotherapy exposure, academic training, global health, non-medical staff, health service infrastructure and recruitment to training programmes were not included.

**Results:**

A total of 146 publications were included in the synthesis. Five themes were apparent through careful iterative analysis representing broadly inter-related issues. Most articles studied radiotherapy training from the perspective of the trainee doctor. Most literature reports results of observational, local or national surveys with a tightly defined scope. Considerable variation exists within hospitals, within countries, over time and between different curricular areas.

**Conclusions:**

Medical education has not kept pace with changes in the field of radiotherapy and large differences are demonstrated in experience between trainees in different hospitals, countries and training stages. Interpersonal relationships, departmental organisation, and national curricula impact on training quality. Qualitative and quantitative research examining modern radiotherapy learning has been uncommon and uncoordinated, until recently. To date no single study has been designed to comprehensively assess a department’s training scheme.

## Background

Clinical Oncology (CO) is the medical discipline encompassing the non-surgical management of cancer, similar to the specialty of Radiation Oncology (RO) in countries such as USA, Canada and Ireland. Physicians in CO treat patients with both radiotherapy and systemic anti-cancer therapy (SACT) however. CO Specialty Trainees are expected to attain mandatory radiotherapy competencies over a period of 5 years in the UK, using a mix of experiential learning, formal postgraduate teaching, self-directed learning and peer learning [1]. Developing skills in radiotherapy relies on workplace-based learning in particular, and CO is a recognised “craft specialty” [2–4] where the apprenticeship model of education is applicable [5, 6].

Over the past two decades, technological advances in radiation beam complexity and in imaging have revolutionised radiotherapy [7]. In working towards the Certificate of Completion of Training, trainees are expected to understand the underlying scientific principles of radiotherapy and become competent in modern treatment planning techniques [1]. Recent trainees have reported significant changes in the learning environment and it is possible that training quality has been adversely affected. Educationalists began to appreciate attrition of the classic apprenticeship relationship across medical specialties and purported this could be deleterious [8].

The aim of this scoping review is to characterise the status quo of the modern training landscape through the identification and synthesis of the existing literature on radiotherapy training. Understanding how trainees learn state-of-the-art radiotherapy will provide a starting point for educationalists with an interest in radiotherapy training, from which to plan future studies and improve local training.

## Methods

### Review technique

This study followed the accepted procedure for scoping reviews [9, 10], and reports results aligned with recommendations by Tricco et al. [11]. The scoping review methodology was chosen rather than classical systematic review for its advantages of being more inclusive and being suited to identifying knowledge gaps [12].

### Research team

The research team comprised a CO Specialty Trainee (GW), and two CO Consultants (also known as Attending Physicians) involved in medical education (ME) (JM) and radiation research (GH).

#### Step 1: definition of the research question

Radiotherapy is a craft wholly learned in the postgraduate setting and medical students’ introduction to the specialty is restricted to an average of 10 h in European medical schools [13]. Training in radiotherapy therefore must include not just the technical nuances of the ever-expanding range of techniques, but also basics, including scientific principles and radiotherapy department logistics. An investigation of what is currently understood about radiotherapy training broadly was undertaken, in order to inform future radiotherapy training studies aiming to improve local standards.

#### Step 2: finding relevant literature.

Two electronic databases (MEDLINE and EMBASE) were interrogated with a search strategy co-designed with a subject librarian on 1st August 2018. Search terms included “radiotherapy”, “oncology”, “education”, “training”, “apprentice”, “postgraduate” and “residency”. Results were restricted to the 20 years after 1998, when modern radiotherapy techniques began to be more widely adopted. Results pertaining to Medical Oncology, workforce trends, undergraduate radiotherapy exposure, academic training, global health, gender disparity, non-medical staff, health service infrastructure and recruitment to training programmes were not included as they were outside the focus of the research. Only papers readily available in English were included. Citation lists were organised with Mendeley software (Mendeley, London, UK). Specifically, reports pertaining to undergraduate education were omitted as it was felt that meaningful radiotherapy training was administered virtually wholly in the postgraduate setting.

#### Step 3: study selection

Following the removal of duplicates, each author screened all abstracts retrieved independently, and rejected those articles not pertaining to the research question. The research team independently reviewed full-text copies of all screened papers to determine their suitability for inclusion in the analysis, based on the criteria listed in Table 1. In total 5 articles were available only as a conference abstract and were excluded, in keeping with scoping review methodology. The team worked collaboratively throughout the review to evaluate and classify articles, with open discussion around cases to reach a consensus where necessary.

**Table 1:** Eligibility criteria for selection of radiotherapy ME publications

Inclusion Criteria	Exclusion Criteria
Study participants were CO/RO Trainees or Consultants.	Study objectives primarily related to non-medical radiotherapy staff.
Study methodology employs qualitative or quantitative techniques.	Studies relating to workforce trends, health service infrastructure and recruitment issues.
Study outcome(s) pertains to the quality of a component of postgraduate training.	Articles relating to equality in training eg gender biases.
Commentary articles or expert reviews.	Publications related to academic training programmes.
–	Studies pertaining to undergraduate training.

#### Step 4: charting the data

The first author created a data extraction spreadsheet using Microsoft Excel (Microsoft, Redmond, USA) and populated demographic, methodological and outcome details. Articles were attributed keywords by one author (and cross-checked by another) to facilitate derivation of themes.

#### Stage 5: collating, summarising and reporting the results

**Fig. 1:**
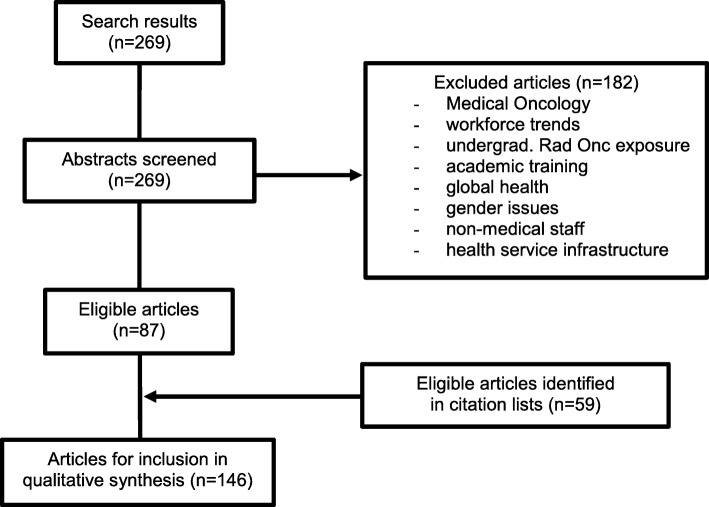
PRISMA flow diagram for selection relevant radiotherapy ME publications

Although not directly applicable to scoping review methodology, Preferred Reporting Items for Systematic Reviews and Meta-Analyses criteria guided the authors’ approach where relevant, and a flow chart of the selection process is included (Fig. 1). A qualitative approach was used to organise the findings, with full-text review of groups of studies preceding summary statements reflecting key messages. This process was carried out by the lead author, and verified by both of the senior authors prior to summarising and reporting the results. Relevant manuscripts were grouped into themes which were felt to broadly represent inter-related issues.

## Results

**Fig. 2:**
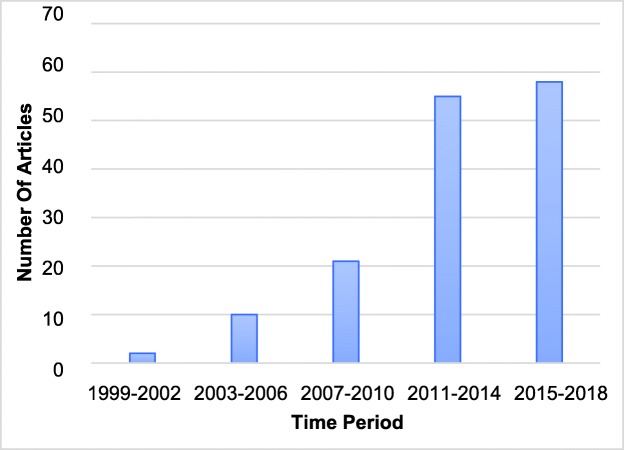
Frequency of radiotherapy ME publications over the last two decades in 4-year bins

**Fig. 3:**
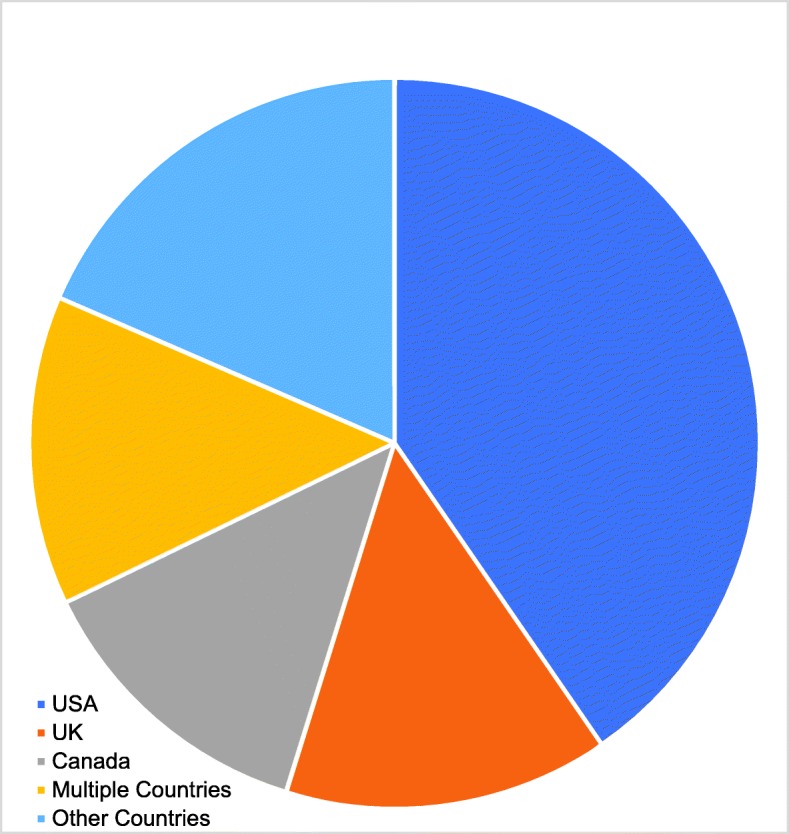
Country of origin of included radiotherapy-related ME papers

A total of 146 relevant articles were identified from the 269 search results. Study designs included surveys (*n* = 62), educational intervention results (*n* = 30), organisation reports (*n* = 28), editorials (*n* = 15), pedagogical investigations (*n* = 8) and interviews (n = 3). A small number of manuscripts featured a combination of these study types. Publications in ME in radiotherapy training have steadily risen in frequency in recent years (see Fig. 2). A minority of countries have published assessments of their radiotherapy programmes and the majority of publications resulted from studies undertaken in the USA. Figure 3 illustrates the proportions of studies from each of USA, Canada and UK, combined efforts two or more between nations, and other countries. Surveys were most frequently published in International Journal of Radiation Oncology*Biology*Physics (*n* = 18), Radiotherapy & Oncology (*n* = 9) and Clinical Oncology (*n* = 7). The mean response rate for published surveys, where reported, was 55% (*n* = 52). Surveys focussed on trainees alone (*n* = 30), trainers alone (n = 9), both trainers and trainees (*n* = 16) or a multitude of radiotherapy-related disciplines (n = 3). Over one third of articles were identified through citation lists. An additional 26 articles were included to introduce relevant background to topics presented. In assessing the available literature, themes emerged as documented with examples below. An exhaustive exploration of the sub-themes is included as a Supplementary Appendix, and this is summarised in Table 2.

**Table 2:** Summary of themes from existing literature base

Themes	Sub-Themes	Definition of Sub-Theme	Example Findings
**Variation in Training**	Inter-role variation	Perspectives on specific issues are role-dependent	There can be a lack of congruence between trainer and trainee accounts of how much time trainees devote to specific activities in some studies
	Progression-related variation	Assessment of quality evolves as trainees become more senior	Trainees may have a larger administrative burden in late training, but improve in confidence of radiology
	Inter-centre variation	Different centres in the same region may have very different resources for ME	Exposure to certain techniques, funding for external training and ‘on call’ burden may vary between centres
	International variation	Resources vary greatly between centres in different countries	Expectations of time spent radiotherapy planning vary considerably between countries and continents
	Temporal variation	The multifactorial nature of training leads to changes over time	Adoption of legislation such as new working hours restrictions, may impact on training delivery
	Activity-related variation	Strong and weak aspects of training co-exist within centres	Trainees report greater competence for common procedures than techniques used infrequently
**Contributing Factors**	Collegiality	Collegiality throughout different levels of the hierarchy improves training	Collegiality amongst peer trainees and seniors contributes positively to learning
	Mentorship scheme	Mentorship is highly valued by trainees	Value of mentors in RO in learning radiotherapy as well as navigating career
	Peripheral units	Create unique learning opportunities but impact on radiotherapy training is uncertain	Less exposure to advanced radiotherapy technologies available outside main cancer hub possibly
	Pre-training experience	Dedicated clinical opportunities are useful for acquiring preliminary principles in radiotherapy	Exposure to oncology-related and radiotherapy clinical scenarios prior to securing formal training post beneficial
	National curricula	Consensus principles for training have been agreed but uptake is variable	Continental and global collaboratives have been set up with the aim of standardising radiotherapy training
	Service provision	Staffing issues directly impact on trainee and trainer educational ambitions	Understaffing is an almost universal problem amongst centres and can affect training quality
	Administration burden	An abundance of low-yield administration is commonly reported by trainees	Trainees in some countries spend up to 10 h per week undertaking activities without any educational benefit
	Job descriptions	A lack of clarity in the expectations on trainees can affect their efficiency and integration	The duality of training and delivering healthcare complicates the definition of clinical responsibilities
	Study-leave budget	Support for educational meetings is not accessible in some institutions	Local/regional policies can restrict some trainees from accessing external training
	Underlying scientific principles	Style of radiobiology and physics teaching impacts on trainee uptake of principles	Inadequate delivery of core radiotherapy principles for building more clinical learning
	Service evolution	Trainee experience is dependent on available radiotherapy techniques during rotations	Local uptake of emerging trends in clinical practice influence the training experience available to trainees
	Trainer-driven curriculum	Involvement of trainees in the organisation of teaching is recognised to be beneficial	International reports have established the gains of involving trainees in the design and delivery of the curriculum
	Economic and political	Training in radiotherapy is not protected from national economic events	Countries have reported disrupted practical elements of training during previous national turmoil
**Impact of Training Quality**	Career progression	Centres where training was undertaken can be important to interviewers for Consultant posts	A graduating trainee’s level of experience carries significant weight at interviews for permanent posts
	Recruitment	Reputation for training quality is associated with competition for training positions	Trainees have been shown to rank posts by the reputed quality of training available at a centre
	Burnout	Poor training quality is associated with increased rates of burnout	Burnout is more likely in TPDs and trainees where there is insufficient time for their respective educational roles
	Fellowship dependence	Trainees may require post-programme training to compensate for inadequate experience	Fellowships may compensate for inadequately covered elements of curriculum or special interests
	Academic aspiration	Academic ambition is reduced in those centres with less emphasis on quality of training	Centres with poorer quality radiotherapy training are associated with less academic aspirations amongst trainees
**Improving Training**	Online training tools	Virtual learning environments are valued by trainees	Online didactic modules and interactive atlases have been shown to be favourable
	Anatomy instruction	Formalised Oncology-orientated anatomy training using scans, lectures and cadavers is effective	Integrated anatomical learning with scans, lectures and cadavers is effective
	Volume delineation lessons	Dedicated contouring teaching is highly sought after by trainees	Small group and webinar-based are moderately effective, common methods of addressing trainee weaknesses
	Trainee societies	Societies provide space for like-minded trainees to benefit from each other’s experience and ideas	Countries with the greatest published outputs in CO/RO medical education have national societies which develop resources
	Simulation	Highly applicable in this technology-centric specialty	This costly educational method suits practical elements of radiotherapy such as brachytherapy
	Logbooks	Mixed views available, depending on format	Logbooks have been championed in surgical specialties, with which RO/CO can be compared in terms of training styles, but are time-expensive
	Leadership training	Increasing emphasis is required in line with other specialties, several model programmes in RO	Online, face-to-face and blended programmes have been established for this increasingly recognised skill in RO/CO clinicians
	Programmatic training	Organised themed sessions favourably ranked by trainees, particularly for rarer clinical scenarios	Integrating seminars, lectures, departmental meetings and electronic alerts over a period of time led to sustained retention of learning
	Applied physics/radiobiology	Practical demonstrations integrated with lectures on challenging principles are successful	A teaching instrument combining practical and theoretical elements of radiobiology and radiotherapy physics has been designed
	Trainee-led continuity clinic	Benefits are available for both trainee learning and patient care due to improved continuity	Patient compliance was increased, trainees reported satisfaction and trainers noted improved workflow and documentation
	Inpatient feedback	Ward-based assessment with immediate feedback involving patient commentary insightful	May improve specifically targeted behaviours amongst trainees and improve trainee satisfaction
	Induction	Meaningful induction required for trainee to gain maximum benefit from a rotation	Trainers may be unaware of induction processes and under rate their value in comparison to trainees
	Device apps	Increasing range of apps available although their clinical validity is often unverified	Apps may be used regularly each day by trainees, especially for more technical tasks such as equivalent dose calculations
**Emerging Pedagogical Themes**	Interprofessional teaching	Mixed group teaching is widely viewed as appropriate and highly valuable	Concurrent training with Radiation Therapists has been shown to be beneficial from the view of both trainers and trainees
	Tailored assessments	Novel, automated, embedded assessment tools are achievable in this technology-centric discipline	Novel planning-based software with integrated feedback components are effective in brachytherapy training
	Near-peer teaching	Content delivered by trainees for trainees is regarded is highly valued	Application of near-peer training in simulation-based learning environments has been successfully undertaken

### Variation in training

This theme was so titled as it encapsulates the shift in expectations and attitudes of trainers and trainees between different contexts, emphasising a lack of standardisation in training provision. Berriochoa et al. found that trainees reported leading more teaching sessions for trainees than reported by trainers and trainees perceived greater benefit from senior-led teaching than peer teaching, in contrast to trainer perceptions [14]. On the whole, trainees increase in confidence in specific competencies as their stage of training progresses eg radiological anatomy [15], although may be more achievable in RO, compared with CO where a trainees’ time is split between SACT and radiotherapy. A survey demonstrated that training opportunities and ‘on call’ burden are better in university hospitals than in non-academic centres and financial support to varying degrees is available to facilitate trainee attendance at professional courses [16]. It was noted that the classification of a centre as academic depends on different criteria in different countries. Whilst most radiotherapy learning (RL) facets vary widely, exposure to plan evaluation is reported as difficult to acquire in most jurisdictions [17–21]. National surveys have demonstrated that perceptions of training fluctuate with time spent in training [22–25] and this pattern was observed in relation to less common tumour sites and emerging radiation techniques in particular. Such trends are likely to be explained by factors including survey learning amongst trainers, expanding access to radiotherapy technology, and organisational changes in postgraduate training nationally including hours rostered.

### Contributing factors

This theme aggregates key areas currently making an appreciable difference to trainers and/or trainees in day-to-day workplace interactions. Collegiality amongst peer trainees and seniors contributes positively to learning [17, 26]. Mentorship has regained popularity in ME literature recently, and the potential value of mentors in RO has been highlighted globally using various methodologies [27–30]. In an attempt to address the discrepancies in radiation-specific Oncology education for trainee doctors, the European Society of Therapeutic Radiation Oncology (ESTRO) designed a curriculum encompassing all essential elements of RL [31, 32]. By its nature, this largely ignores competing interests in SACT for CO trainees. European collaborators proposed guidelines for staffing, training resources and educational infrastructure but despite endorsement by the European Radiotherapy Board, guideline adherence has not been audited [33]. Service provision not only impacts trainees, but it has an effect on the performance of TPDs [34]. A European study reported greatest level of trainee agreement in relation to ‘less bureaucracy’ as a method of improving their RL [16]. The increasing technical complexity of radiotherapy and the highly specialised skills required by operators has meant that the uptake of many technologies is variable between centres [35]. Conventionally trainee involvement in the design of Specialty Training has not been routine, but the promotion of trainee opinion to provide constructive feedback has been recommended [36–38].

### Impact of training

This section explore the downstream effects associated with these factors. Clinical training quality may be the leading factor in preference of RT centre for trainees, and in the context of a post deficit, better trainees may be recruited to centres with relatively better training quality [39]. A study of TPDs found correlation between a lack of time for educational activity and emotional exhaustion [34]. Survey-based evidence suggests that Fellowship training is used to compensate for inadequate experience during Specialty Training in areas of the curriculum, in addition to their intended purpose of gaining skill not accessible in their training [40].

### Improving training

This theme summarises the available evidence of approaches incrementally improving specific aspects of training. Cohort studies of trainees exposed to an integrated programme of radiological, didactic and cadaveric demonstrations found that both knowledge and contouring performance were significantly improved [41–43]. RL comprising an interactive, small-group contouring demonstration following a didactic lesson in radiological anatomy of the head and neck was feasible and effective in the transfer of skills [44]. For gynaecological brachytherapy training, supervised trainee-performed clinical procedures have been followed up with simulated practice at a delayed timepoint by investigators [45]. Scrutiny of practical skill and subjective confidence level appeared to validate this competency-based approach. Modular teaching relating to leadership skills in the realm of RL have been successfully trialed [46]. ‘Continuity of care’-centred clinics operated by trainees have been successfully instigated in a US cancer centre, with observed benefits for both trainees and patients [47]. Such approaches may have more benefit for RO trainees who aren’t usually involved in SACT, unlike CO trainees. A survey of trainer and trainee views regarding orientation revealed differences of opinion, for example, trainers were more likely to think trainees were ready to begin clinical work on day one, and trainers believed that a formal introductory curriculum was in place at their centre when it wasn’t [48]. Trainers were less likely to believe that a formal induction made a trainee better prepared for clinical work; trainers and trainees had very different levels of agreement regarding the desired content of a useful introductory module. Another option considered by these investigational educators was off-site ‘boot camp’ training, which is commended for the removal of clinical distractions and increasing the social context of orientation.

### Emerging pedagogical themes

‘Emerging Pedagogical Themes’ collects the educational paradigms on the horizon that seek to enhance training experience. Given the interprofessional collaboration involved in the patient radiotherapy journey, the feasibility and effectiveness of training doctors alongside Physicists and Radiation Therapist could be deemed unsurprising [4, 49–52]. Statistical techniques were used to create a competency checklist for gynaecological brachytherapy [53] and advanced qualitative methodologies produced consensus target volumes for assessment of contours [54]. Near-peer teaching has been applied in CO informally historically, but also in newer contexts such as simulated and interprofessional training [51].

## Discussion

Qualitative and quantitative research examining modern RL is limited. The senior-led decision making nature of Oncology has engendered an educational model of apprenticeship, where trainees learn through clinical encounters and technical aspects of RO shared with their clinical supervisor. As the apprenticeship model has become less suitable with the evolution of clinical radiotherapy, witnessed also in other medical disciplines [8] so clinical training requires renewed investment to protect training standards. This study has identified a surge in ME publications in CO and RO since 2011. A majority of the available literature reports findings from observational, local or national surveys with a tightly defined scope. Most articles were surveys of the trainee’s perspective only. Five themes were apparent through careful iterative analysis representing broadly inter-related issues.

### Limitations

The unsupervised element of this scoping enquiry, chosen to capture as much of the wide-ranging and separate relevant literature as possible, means that emerging themes received relatively equal scrutiny. More focussed future studies could increase the depth of the analysis of many areas, with a greater availability of evidence as it emerges. Whilst other medical specialties undergoing evolution may reconcile with some of the identified factors, it is likely that content specific to RO/CO will limit the applicability. Furthermore, there may be differences in how the learning from this study can be applied between the CO and RO specialties, and articles relating to the interaction between radiotherapy and SACT training were not included in this study. Included studies were not subject to a rigorous assessment of study quality. This is an expected disadvantage of scoping review methodology, where data from a wide range of sources are accessed. The exploratory nature of the scoping review was felt to be justified given the lack of previous review papers. In addition, a small number of articles were noted to cover several areas, and it is anticipated that some areas were explored more than others during the synthesis. Over one third of articles were not identified directly in the search implying the search terms for future overviews should be carefully optimised in this diverse, expanding field. Initiatives existing purely in the ‘grey literature’ are likely to have been missed as this was not a traditional systematic review.

While RO and CO disciplines may be regarded as very similar with respect to clinical practice, there may be significant international variation in the circumstances of ME, in terms of policy, resource and attitude. Furthermore, there could be subtle intercontinental differences in the nomenclature and the predominant representation of USA, UK and Canada may restrict the applicability of results to other countries eg definition of academic centres. However, institutions which have not yet assessed their training may learn from the published experience of institutions which are actively developing their training programme. Articles identified and the results generated pertain virtually entirely to high income countries, restricting the applicability of this scoping exercise. It is anticipated that the global shortage in radiotherapy facilities and staffing in lower- and middle-income nations amplifies the challenges outlined in this study. Lastly, this study fails to address the impact of training in systemic therapy on the junior doctor’s RL in those countries where RO is incorporated within CO training, compared to those in which it is not.

### Proposals

The collated literature on contemporary CO ME has not been published previously. The scope of identified studies in this review capture elements of training quality, its determinants and impact, and both existing and novel methods of change. The authors’ recommendation is that national training bodies should work towards the development of a consensus around ideal minimum standards for local training circumstances. As a minimum, additional formal ME resource will be required, for example, trainee and trainer ME Fellowships, support for postgraduate qualifications and practical resource such as journal subscriptions.

Topics could each serve as individual foci of study, which will be warranted for more complex issues. eg. simulation in RO/CO ME. Coordination of studies through purpose-built national networks would facilitate concerted efforts on a large scale, creating a more robust dataset and increasing the impact of outputs. Equally, the themes drawn out from the literature in this study could represent a reasonable grounding for a five-pronged approach which training programme directors evaluating local RO/CO training could centre data collection around, for example:

- Variation in training – ‘is the training standardised between centres / teams / doctor grades?’
- Impact of training quality – ‘how many trainees get Consultant posts / burnout / academic roles?’
- Contributing factors – ‘do we have a mentorship programme / study leave / trainee involvement?’
- Improving training – ‘do trainees have a society / log book / adequate induction / online tools?’
- Emerging pedagogical themes – ‘do we have novel local solutions for improving training?’

Although the paucity of CO ME research is being addressed globally, without a research network and framework, most facets of clinical training have not been thoroughly examined. ME research designs available to researchers include observational and interventional, and both qualitative and quantitative techniques. As outlined in the results, successful qualitative approaches include interviews, focus groups and questionnaires, and these add layered complimentary insights to familiar quantitative approaches such as questionnaires. It is anticipated that trainer and trainee perspective of local factors should guide the local study designs. The authors would beckon that efforts are made to publish all evaluations and initiatives in RO/CO ME to ensure findings can be included in future overview exercises. The welcome recent increase is probably secondary to improved awareness of the value of ME research, particularly through research collaboratives such as the Radiation Oncology Education Collaborative Study Group in the USA and curriculum development frameworks such as CanMEDS in Canada.

No studies have attempted to comprehensively assess a department’s clinical training and it is likely that methodology such as institutional ethnography would be particularly applicable [55]. This technique is used in healthcare professional research to analyse the inner workings of a system of people linked by a common goal, using an “on the ground” approach. Institutional ethnography moves away from abstract hypotheses by encouraging the researcher to access people’s actual practices, and it makes the case for reflexive learning about a system from an internal viewpoint as opposed to an external account. A smaller scale, ‘mixed methods’ approach may also reveal intrinsic factors [56]. In an increasingly pressurised system, it would be prudent to optimise mechanisms of CO ME to protect the strengths and build on weaknesses, proactively ensuring clinical training infrastructure prevails during the threat of service-driven reduced momentum.

## Conclusions

ME has not kept pace with the rate of change in the field of radiotherapy and there are large differences in the experience of trainees in different hospitals, countries and training stage. Interpersonal relationships, departmental organisation, national curricula and government policies impact on training quality. Societal reports and interventional studies acknowledge these disparities and have proposed action plans. Further focussed research is urgently required to maintain training quality in this ever-moving clinical discipline.

## Supplementary information

**Additional file 1.** Supplementary index.

## Data Availability

Search strategy defined in methods.

## Abbreviations

CO: Clinical Oncology
RO: Radiation Oncology
SACT: Systemic Anti-Cancer Therapy
M: Medical Education
