# Supplementary material for: Learning radiotherapy: the state of the art
Source: BMC Med Educ. 2020 May 11;20:150. doi: 10.1186/s12909-020-02054-z (PMC7216702; doi:10.1186/s12909-020-02054-z)
Supplement: Supplementary file 1 — Additional file 1. Supplementary index. [file 12909_2020_2054_MOESM1_ESM.docx]

**Supplementary Index**

**Variation in training**

*Inter-role variation*

Perceptions of quality of radiotherapy training are dependent on the status of the observer. A survey-based study of Radiation Oncology clinicians identified that trainers and trainees report different levels of engagement with certain activities eg assessing new and review patients, supervision in clinics, representation at multidisciplinary meetings and target delineation (1). For each of these learning opportunities trainee responses indicated lower trainee participation than trainer responses. RO clinicians have different opinions on isolation in the radiotherapy department according to role (2). Compromise of protected teaching by clinical duties was more prevalent according to trainees, and trainers described conducting mock oral examinations more than trainees reported receiving these. Trainee exposure to stereotactic radiotherapy is regarded as crucial in studies of both trainers and trainees yet current standards of training as assessed by trainers and trainees are inconsistent (3,4). In contrast, other studies of trainers and trainees identified concordance in most categories of trainee performance, with the exceptions of head and neck treatments and plan evaluation (5). Agreement was also observed between trainers and trainees in domains such as the superior efficacy of interactive, question-based and small group teaching over didactic styles (6), involvement in planning scans and treatment verification (1), side effect management (5), and evidence-based discussion for supervised clinical encounters (6).

*Progression-related variation*

Levels of satisfaction reported by trainees vary with progression through training. Survey responses from senior US trainees indicated increasing hours of work per week on average compared with junior counterparts (7). In this study trainees reported that they spent increasing time completing tasks that could be completed by non-clinical colleagues as they progress through training programmes, and that the extent of this problem increased over a three year period (7). UK trainees commonly spend time out-of-hours gaining radiotherapy experience such as volume delineation due to lack of opportunity during working hours, related to the aforementioned administrative responsibilities (8). A study noted that trainees have progressively more positive reflections of their pre-residency Oncology experience (9).

*Inter-centre variation*

Comprehensive regional studies in other countries elicited disparity between centres in the same network regarding radiotherapy planning opportunities for trainees (10). Educational researchers North America reported peripheral unit placements in half of registered training programmes (11). Other surveys suggest trainees do not have equitable access to protected educational time across the national programme (8,12). Another survey indicated a trainee preference for watching and then doing, quizzing, regular one-to-one and shadowing but the uptake amongst trainers varied between centres (13). A survey of new Consultants demonstrated that very limited time is allocated to education in job plans and a minority of their clinical sessions involve trainees (14). Despite these findings, approximately half of the participants accepted official education duties as supervisors, for which a minority were provided supporting professional activities time (14), which is a recognised obstacle to completion of workplace-based assessments (15).

*International variation*

Reports that UK trainees typically attended two or fewer radiotherapy planning sessions per week raised concern (16). Contemporaneous European recommendations are that clinicians delivering radiotherapy devote a minimum of 60% working hours to radiotherapy (17). Australian ROs have recently observed an improvement in the balance of workload, most likely owing to greatly increased oncologist and trainee numbers (18). Specific guidelines for planning of trainee posts vary greatly between EU countries (19) and between the EU, Canada and Australia (20). Common international goals with respect to protected teaching time and resources do exist (21,22). Just one survey found satisfactory outcomes in this area (23). Similarly, virtually all trainees at European RT centres ranked practical clinical training as ‘most important’ or ‘very important’ for effectiveness of teaching in a Europe-wide survey (19).

*Temporal variation*

Organisational changes in postgraduate training nationally are prominent in the UK landscape for example, which now has a formalised competency-based assessment system, in contrast to the previous apprenticeship model where trainees often proceeded through training without annual appraisals (16). Published historical perspectives document the absence of high quality radiotherapy learning (RL) for trainees during the expansion of Oncology services in the 1990s (20). The importance of partnership between trainees and trainers in RL has been reaffirmed recently (10,24).

Several studies depict specific trends in training, for example, an increase in the use of lecture-based learning (25) and increasing competition for training Fellowships (26). Interval surveys have also demonstrated that weak areas may fail to improve over time, such as the ratio of service provision to education (27). Similarly, trainee survey results regarding theoretical courses for examination preparation have demonstrated persistent weaknesses (8). With significant innovation in radiotherapy delivery (28), surveys depict how teaching of emerging techniques such as IMRT evolved as more centres embraced the new technology (29–31). Technical changes and the dynamic available skill mix in radiotherapy departments prompted the revision of postgraduate curricula over time (32). The maturation of radiotherapy Peer Review meetings in recent years offers trainees a new structured resource for RL (33) and UK surveys substantiate its popularity (34). RL has also been boosted in many centres by reducing compromise of radiotherapy activities by inpatient clinical care via introduction of a rotating ward trainee (8,14,34,35).

*Activity-related variation*

Theoretical competency has been self-evaluated positively in a majority of domains relating to radiotherapy, though areas of weakness were identified in radiobiological modelling and treatment planning software (5). A survey of US Training Programme Directors (TPDs) demonstrated agreement that palliative radiotherapy is an important treatment paradigm, and a formal curriculum was in place to support the development of such skills in a majority of centres (36). In the absence of a dedicated palliative radiotherapy clinic, trainees gained experience during their rotations including ‘on call’ (37). Obstacles to effective training in palliative radiotherapy were not identified in this study, though they have been characterised in published anecdotal reflections (38). Dietzel et al showed that knowledge self-evaluation was impressive for more common techniques and procedures, but poor for more sophisticated or less common activities (30). There is considerable literature on the difficulties of brachytherapy training due to infrequent practice amongst ROs (39–41). Amongst Canadian trainees, approximately three-quarters believe geriatric Oncology has not been incorporated into the curriculum sufficiently (42). A separate survey found radiology teaching to be suboptimal and that teaching from a Consultant would improve confidence (43). Of equal impact, broader topics may be completely omitted from programmes, for example patient safety and quality improvement training (44), and management of the radiotherapy department (45). Blended learning techniques for trainees regarding high quality radiotherapy practice have proven successful, integrating seminars, lectures, departmental meetings and electronic alerts (46). In adopting the exponent of radiotherapy technology it is essential for trainees to retain focus on holistic, safe patient care (47).

**Contributing Factors**

*Collegiality*

An Australasian study found that organised teaching provided the opportunity for social support (48). Awareness of a positive working environment and high levels of morale are frequently-listed determining factors in career and hospital choice (8,19,48–51).

*Mentorship*

A model of close working between trainer and trainee was previously commonplace for trainees learning radiotherapy (16) and trainee representatives have called for renewed trainer commitment (11). Increasing mentorship was prioritised as a future workstream at The Second Intersociety Radiation Oncology Summit (52).

*Peripheral units*

Centralised staff required for cancer care travel to peripheral clinics for patient convenience globally, and surveys of American trainees suggest rural activity provides unique learning opportunities (7)**.** In countries where there is joint responsibility for systemic and radiation therapies, experience at peripheral units may offer little radiotherapy exposure. It is not known if this impedes the trainee’s familiarisation with the pertinent repertoire of radiotherapy skills.

*Pre-training experience*

Exposure to radiotherapy prior to formal training has been rated highly by trainees, and experience with patients in the inpatient setting was crucial to their maturation as an RO clinician (9). Suboptimal undergraduate radiotherapy teaching is a topical, separate research focus (53).

*National curricula*

Transatlantic collaboration unified the international expectations of future ROs for a range of professionals involved in radiotherapy including clinicians (54). Previously efforts to standardise RL across Europe have been met with limited success (19). National and international inter-group curricula have been agreed pertaining to the physics content of RO medical training, including adaptation by postgraduate examination boards (55–57). Such studies have been designed robustly and iteratively with a Delphi study design. Topical and specialised areas such as protons have dedicated curricula to help trainers deliver effective programmes (58–60). A global consortium has been formed with the aim of analysing how resources and assessment tools could be shared internationally (61).

*Service provision*

Issues regarding staffing ratios are seemingly universal as high quality training programmes also report understaffing (30). Several European countries are experiencing a multifactorial recruitment problem and projections for the next decade suggest patient safety may be jeopardised (62–65). Published evidence and consensus statements warn of the link between poor standards of training and inadequate staffing (66). In the USA there is an abundance of trainee doctors in RO programmes, the opposite dilemma from the UK (67). Canadian training programmes have recently produced an excess of qualified trainees but extrapolations of predicted retirements, part-time working trends and population disease trajectories indicate an impending staff deficit (20). Comparable results have been found in studies of estimates for increasing cancer survival, technically advancing radiotherapy and rising clinical trial access (68).

*Administration burden*

Almost half of US trainees spend more than 10 hours completing activities that they perceive to be of no educational benefit weekly (11). Moreover, a study of new Consultants found that training didn’t provide adequate preparation for the non-clinical component of their post (40).

*Job descriptions*

The duality of a trainee position, fulfilling responsibility to deliver healthcare and simultaneously striving toward learning objectives, complicates the characterisation of the role (69). The differences between trainer and trainee views with regard to the activities of trainees in their final year have been highlighted (69). Advice is provided for TPDs on the design of UK trainees’ weekly timetables, which are intended to mirror their Clinical Supervisors’ schedule (70,71). Such tools provide a framework for how the parallel roles of a trainee may co-exist synergistically but implementation is challenging (8,10,15,35). Another trainee role issue is the legal responsibility and therefore engagement in radiotherapy is limited by lack of accreditation, both in relation to assertiveness and inclusion in team-based activities (64).

*Study-leave budget*

National meetings provide a valuable opportunity for learning by trainees, and many training programmes financially support attendance (15) but trainees don’t receive equal resources owing to local/regional policies (34,72). US studies have shown the significantly different perspective of this problem from trainers and trainees (69) and how some trainees are not aware of their entitlements (8).

*Teaching of basic principles*

The principles underlying radiotherapy are essential for practice and examinations. Courses comprise of condensed one-week modules, rolling schemes of regular sessions, and workplace-based intensive blocks of exposure (73). The subject matter complexity justifies the existing focussed training interventions, however there is an absence of consensus in debated areas of the curriculum (74). Several nations have reported a recent reduction in the delivery of radiobiology and physics by specialists because of the diversification of young radiobiologists into other areas of science (75,76). Signals from trainee surveys highlight an increasing disconnect between delivered teaching and examination curricula (34,75).

*Service evolution*

Brachytherapy training has become difficult to obtain in some countries due to new trends in clinical practice (39), often due to competing training demands (77). Many trainees have limited access to experiential learning with modern imaging, relying solely on infrequent one-to-one teaching from their trainer, with less frequent input from a Radiologist (78).

*Trainee-driven curriculum*

An interventional teaching study demonstrated how trainees would alter specific areas of their programme to improve it (79). Since the early 1990s trainees surveys have helped provide training programme coordinators with feedback from the front line (16,80–82). One aspiration of the ESTRO School is to incorporate trainees into the management of the School to enhance the utility of their resources (83).

*Economic/political*

In some countries, ongoing or recent political factors have negatively impacted on the development of modern radiotherapy services, and this negatively impacts training (84,85). Strategies to improve RL in such circumstances have been proposed (86).

**Impact of training quality**

*Career progression*

Analyses of the attributes that Consultant Clinical and Radiation Oncologists desire in future colleagues when interviewing candidates suggest that a graduating trainee’s level of RT experience carries significant weight (87). The same authors found that the training programme to which a trainee belonged was also influential in the hiring decisions.

*Recruitment*

Caution should be applied however as perception of training quality at a centre may not be representative. It has been found that introductory courses to a training programme for undifferentiated doctors can be used to improve recruitment (88).

*Burnout*

RO is an emotionally charged discipline of medicine and several large studies have shown that burnout, a syndrome of ﻿emotional exhaustion, depersonalization and low personal accomplishment, is common amongst trainees in many countries (89–92), and amongst trainers (93). Quality of medical training was identified as a factor in two studies (89,91). As correlation was found between a lack of time for educational activity and emotional exhaustion for TPDs, it may follow that increased time for education, amongst other interventions, could reduce the incidence of burnout (94). One example of how this principle can be translated in practice is procedural competency, where improvements in trainee confidence in their radiotherapy skills are observed following brachytherapy coaching with trainers (95). A separate study revealed the importance of regular trainer feedback in level of satisfaction for trainees (96).

*Fellowship dependence*

Many trainees in RO/CO seek a post-training clinical Fellowship prior to application for a permanent position to further their experience in a particular aspect of radiotherapy. This method of recoupment of skills may be deemed harmful given that it doesn’t include an inherent mechanism for addressing weaknesses of a training programme, and because reliance on Fellowships is probably not sustainable given rising competition (89). Furthermore, many trainees do not utilise their Fellowship skills in the long-term (97).

*Academic aspiration*

Learning environments with less time devoted to teaching of radiotherapy skills are associated with reduced academic aspirations amongst trainees (98). Participation in academic activity has been shown to have a positive effect on trainees (99).

**Improving training quality**

*Online training tools*

Interactive web-based atlases have been developed for use by trainees when contouring areas of interest on planning scans (100). Preliminary results of a multi-institutional validation study are promising, with usability and satisfaction both rated highly, an improvement in clinical target volume (CTV) and ‘organ at risk’ delineation, compared with standard resources. Online modules with linkage to treatment planning concepts were rated highly by users (101). Didactic webinars targeting trainee-reported curriculum areas of difficulty have proven successful, exemplifying trainee-centred postgraduate RT training and the acceptance of web-based tools (102). Virtual training instruments blended with ‘face-to-face’ teaching have been utilised for RT training (83).

*Anatomy instruction*

With image-based developments such as MRI, IGRT and 4D-planning in radiotherapy, it is thought that the disciplines of RO and Radiology are converging (103). Multidisciplinary teaching has proven popular with trainees (104) and has been a long-standing strength of one programme (105–107). In addition, this integrated course was associated with durable learning, and high trainee satisfaction (108).

*Volume delineation lessons*

RL comprising an interactive, small-group contouring demonstration following a didactic lesson in radiological anatomy of the head and neck was feasible and effective in the transfer of skills (109). This small study focussing on short-term outcomes found significant improvements in CTV delineation independent of stage of training. Conversely, dosimetric analysis of a thoracic contouring teaching intervention revealed minimal improvement although the investigators tested groups rather than individuals, and pre-test performance was considered excellent (110). A Canadian study of a teaching intervention for contouring of prostates also revealed improvements, albeit non-significant differences in the small, heterogenous population (111). Over half of experimental arm subjects stated that they would use their experience as a basis of change in everyday practice. In tandem with real-world practice, studies testing the validity of auto-segmentation software incorporate trainees (112). Although usability and efficacy as a training tool have not been determined, the trainee willingness for involvement in emerging technologies is clear. Fellowship in Anatomic delineation and CONtouring (FALCON) is a widely used digital platform on which to gain contouring skills and this project headed by ESTRO School has had success at national meetings and for individuals (113).

*Trainee societies*

Regional trainee-orientated groups, often nested within an established national society and coordinated by panels of trainees and overseeing trainers, provide a popular framework for learning, mentoring and networking (114). The New York Roentgen Society is devoted to these objectives and has an acute awareness that its purpose is not didactic scientific teaching. Conversely, the young French trainees’ society organise a teaching curriculum and annual educational summer school (115). They have also developed virtual resources for trainees such as radiobiology apps and web-based radio-anatomy resources. A similar German society has been formed with the aim of increasing training quality and national society involvement of young academics (116). The Italian equivalent’s projects have included clinical teaching, fostering effective presentation skills, and topical non-clinical and clinical surveys (117). In the UK, the Oncology Registrar’s Forum (ORF) is an elected group of trainees recognised by RCR tasked with representing trainee interests and connecting them to RCR (118). Training collaboratives may also centre on one particular function, for example trainee research in the UK (119).

*Simulation*

Contemporary undergraduate and, increasingly, postgraduate medical training combines classical methods with simulation-based teaching, and it has been shown that this model can be applied to RL (120). In addition to simulating real-world settings for maximal learning for junior trainees, this construct provides both supervisory experience and reinforcement of working relationships with non-clinical staff for senior trainees. Simulation has also been successfully applied in the setting of practical procedures such as dosimetric analysis of brachytherapy seeds in a simulated phantom to provide evidence for competency-based assessments (121). The major advantages of simulation-based training over traditional teaching styles are the two-way communication during feedback and the option for rapid iterative feedback-based learning (122). The drawback of relatively high cost in terms of time and consumables is acknowledged by simulation-based educators (123).

*Logbook*

Assessment of trainee activity and competency in several disciplines requires maintenance of a logbook. Whilst the UK CO training programmes are based on a wider competency assessment platform, many countries in Europe require trainees in RO to complete a logbook of all completed radiotherapy cases (124). Logbooks were written into legislation that permitted doctors qualifying within the EU to work anywhere inside it in the 1980s. Recommended contents included theoretical and clinical training course attendance, clinical experience, presentations and publications. The benefits of a logbook include facilitation of training quality standards benchmarking as well as individual trainee competency evidence. The challenges of logbooks are recognised to be the optimisation of the format for efficient and safe population, and acquiring ‘buy in’ of trainers and trainees who are already over-stretched (34).

*Leadership training*

Encouraging trainees to take responsibility for their work and learning has featured as an important concept in several surveys of new consultants (14,125). Trainee engagement in selection of content for local teaching programmes and organisation of delivery has proven to be successful (126). Modular teaching relating to leadership skills in the realm of RL have also been trialled (127). A pilot study of an online leadership training tool for use on trainees’ handheld devices has demonstrated its feasibility (128).

*Programmatic training*

Combining techniques with a single theme have been tested in trainees, regarding safe and high quality RT practice (46). Integrating seminars, lectures, departmental meetings and electronic alerts over a period of time improved knowledge with time and led to sustained retention of learning, for trainees in RO, and other staff groups. This approach to knowledge transfer can overcome barriers of traditional methods, which are known to have limited application when striving for long-term retention, and in particular regarding less commonly occurring topics (129).

*Applied physics/radiobiology*

Recognising the dearth of resources for senior trainees preparing for examinations and Consultant practice, one US university developed a teaching instrument integrating both theory and practical elements of RO (130). It is anticipated that this will enhance assimilation of the theoretical principles of physics and radiobiology, and that practical content will boost the potential gain from such solutions.

*Trainee-led continuity clinic*

‘Continuity of care’-centred clinics operated by trainees have been successfully instigated in a US cancer centre, with observed benefits for both trainees and patients (131). Patient compliance was found to be increased, trainees reported improved satisfaction with their educational experience and trainers noted both a smoother workflow and augmented documentation compared with Consultant-led models.

*Inpatient feedback*

Application of ‘audit with feedback’ on trainee performance on the RO inpatient ward had positive consequences on RL, congruent with observed effects in other medical specialties: improved targeted behaviours amongst trainees and high levels of participant satisfaction (132). ‘Audit with feedback’ is an educational proforma where trainers regularly inspect trainee performance against accepted standards and provide constructive feedback.

*Induction*

Many trainees struggle with the sudden-onset immersion in the world of radiotherapy following a successful post-qualification experience in general medicine or surgery (133). Almost half of US trainees surveyed believe an orientation is essential, and a minority of those view the orientation received as valuable (134). Duration of the orientation period was shown to be correlated with trainee-perceived helpfulness. A re-invented orientation programme markedly increased UK trainee agreement on the quality of the induction for the radiotherapy treatment planning system, radiotherapy planning workflow and departmental contacts and lay-out (135). National advice regarding induction has been published in the UK (136).

*Electronic devices*

As with other areas of medicine, apps have been designed for a variety of tasks performed by radiotherapy clinicians (137). Popular apps have been interrogated with a scoring system based on literature base, financial cost, UK alignment and degree of currency. Journal-based and guideline apps were highly rated. Drug manual apps performed poorly due to expense. It is noted that there is no regulatory body to which app designers are responsible. The available apps relevant for radiotherapy have been mapped in a systematic review by Spanish investigators (138). A study of French trainees found that virtually all trainees used a smartphone in the course of their professional duties five or more times per day (139). Two-thirds used them for radiotherapy ‘equivalent dose’ calculations. Alarmingly only half of trainees checked the validity of their apps. There are no studies investigating the use of apps by trainers, and study participants in the latter study were more likely to be younger trainees.

**Emerging Pedagogical Themes**

*Interprofessional teaching*

Further justifying the positive signals observed in interprofessional interventions, opinion survey results display a high level of agreement amongst radiotherapy staff regarding knowledge gained from interprofessional training (140,141). Surveys of trainers and trainees consistently indicate that involvement of multiple disciplines is advantageous for learning (30,140,141).

*Tailored assessments*

Conventional reproductive learning and behaviour-centred models of education focussing on the content and the teacher have been superseded by learner-centred, constructive and learning outcomes-based education. Concomitant novel methods for assessing trainees are therefore required, and these are in development for practical and theoretical curricular items (142,143). The technology-driven basis of their specialty is likely to promote the engagement of RO clinicians with novel assessment techniques, and with the efficient, objective assessment of trainee performance (112). Competency-based assessment of trainees has been adopted in medical curricula globally and this format is applicable for radiotherapy skills (144).

*Near-peer teaching*

This style of teaching is defined as education delivered by trainers one or more years senior to the trainees, but within the same broad grade (145). Pilots of this technique enjoyed success for both junior and senior participants in the general medical setting (146). Further refinement and robust support for near-peer teaching in RL may enable departments to cope with reduced time for training, whilst providing valuable teaching and leadership experience for more senior trainees.

**REFERENCES**

1. Thureau S, Challand T, Bibault JE, Biau J, Cervellera M, Diaz O, et al. Delegation of medical tasks in French radiation oncology departments: Current situation and impact on residents’ training. Cancer/Radiotherapie. 2013;17(5–6):370–7.

2. Holt T, Bydder S, Bloomfield L. Survey of the learning activities of Australasian radiation oncology specialist trainees. J Med Imaging Radiat Oncol. 2008;52(6):605–10.

3. Sheehan J, Suh JH, Kavanagh B, Xu Z, Ren L, Sheehan K, et al. Training Neurosurgery and Radiation Oncology Residents in Stereotactic Radiosurgery: Assessment Gathered from Participants in AANS and ASTRO Training Course. World Neurosurg. 2018;109:e669–75.

4. Samuel N, Philteos J, Alotaibi NM, Ahuja C, Mansouri A, Kulkarni A V. Canadian Neurosurgery Educators’ Views on Stereotactic Radiosurgery in Residency Training. World Neurosurg. 2018;112:e208–15.

5. Franco P, Ciammella P, Peruzzo Cornetto A, De Bari B, Buglione M, Livi L, et al. The STYRO 2011 project: A survey on perceived quality of training among young Italian radiation oncologists. Med Oncol. 2013;30(4).

6. Berriochoa C, Weller M, Berry D, Reddy CA, Koyfman S, Tendulkar R. Program director and chief resident perspectives on the educational environment of US radiation oncology programs. Pract Radiat Oncol. 2017;7(1):e65–70.

7. Gondi V, Bernard JR, Jabbari S, Keam J, De Amorim Bernstein KL, Dad LK, et al. Results of the 2005-2008 association of residents in radiation oncology survey of chief residents in the United States: Clinical training and resident working conditions. Int J Radiat Oncol Biol Phys. 2011;81(4):1120–7.

8. Kosmin M, Brown S, Hague C, Said J, Wells L, Wilson C. Current Views on Clinical Oncology Training from the 2015 Oncology Registrars’ Forum Survey. Clin Oncol. 2016;28(9):e121–5.

9. Baker SR, Romero MJ, Geannette C, Patel A. The Value of the Internship for Radiation Oncology Training: Results of a Survey of Current and Recent Trainees. Int J Radiat Oncol Biol Phys. 2009;74(4):1203–6.

10. Wong DW, Sanghera P, Stevens AM, Grieve RJ. Survey of West Midlands Clinical Oncology Trainees. Clin Oncol. 2008;20(1):98.

11. Nabavizadeh N, Burt LM, Mancini BR, Morris ZS, Walker AJ, Miller SM, et al. Results of the 2013-2015 Association of Residents in Radiation Oncology Survey of Chief Residents in the United States. Int J Radiat Oncol Biol Phys. 2015;94(2):228–34.

12. Leung J, Munro PL, James M. Faculty of Radiation Oncology 2014 workforce census. J Med Imaging Radiat Oncol. 2015;59(6):717–27.

13. Yee D, Fairchild A, Keyes M, Butler J, Dundas G. 2003 Survey of Canadian radiation oncology residents. Int J Radiat Oncol Biol Phys. 2005;62(2):526–34.

14. Dickson J, Liu D, Bloomfield D. Training in Clinical Oncology and the Transition from Trainee to Consultant: Results of the Royal College of Radiologists’ 2015 Post-Certificate of Completion of Training Survey. Clin Oncol. 2017;29(3):e64–71.

15. Said J, Woolf DK, Glendenning J, Leaning DJ, Manetta C. The current views of clinical oncology trainees. Clin Oncol. 2014;26(3):159–61.

16. Nutting C, Short S. Where now with training in clinical oncology? Clin Oncol. 2001;13(1):4–5.

17. Baumann M, Leer JWH, Dahl O, De Neve W, Hunter R, Rampling R, et al. Updated European core curriculum for radiotherapists (radiation oncologists). Recommended curriculum for the specialist training of medical practitioners in radiotherapy (radiation oncology) within Europe. Radiother Oncol. 2004;70(2):107–13.

18. Leung J, Vukolova N. Faculty of Radiation Oncology 2010 workforce survey. J Med Imaging Radiat Oncol. 2011;55(6):622–32.

19. Bibault J-E, Franco P, Borst GR, Van Elmpt W, Thorwhart D, Schmid MP, et al. Learning radiation oncology in Europe: Results of the ESTRO multidisciplinary survey. Clin Transl Radiat Oncol. 2018;9:61–7.

20. Stuckless T, Milosevic M, De Metz C, Parliament M, Tompkins B, Brundage M. Managing a national radiation oncologist workforce: A workforce planning model. Radiother Oncol. 2012;103(1):123–9.

21. Jani AB, Marshall D, Vapiwala N, Davis SB, Langer M. Results of the 2014 Survey of the Association of Directors of Radiation Oncology Programs (ADROP). Pract Radiat Oncol. 2015;5(6):e673–8.

22. Benstead K, Turhal NS, O’Higgins N, Wyld L, Czarnecka-Operacz M, Gollnick H, et al. Multidisciplinary training of cancer specialists in Europe. Eur J Cancer. 2017;83:1–8.

23. Malik R, Oh JL, Roeske JC, Mundt AJ. Survey of Resident Education in Intensity-Modulated Radiation Therapy. Technol Cancer Res Treat. 2005;4(3):303–9.

24. Gwynne S, Gilson D, Dickson J, McAleer S, Radhakrishna G. Evaluating Target Volume Delineation in the Era of Precision Radiotherapy: FRCR, Revalidation and Beyond. Clin Oncol. 2017;29(7):436–8.

25. Gondi V, Bernard JR Jr, Jabbari S, Keam J, de Amorim Bernstein KL, Dad LK, Li L, Poppe MM, Strauss JB CCAEC. Results of the 2005 to 2008 Association of Residents in Radiation Oncology surveys of chief residents in the United States: didactics and research experience. Am J Clin Oncol. 2012;35(1):32–9.

26. Mohamad O, Meyer JJ. Recent Trends in Radiation Oncology Fellowship Training in the United States. Int J Radiat Oncol Biol Phys. 2017;99(3):539–40.

27. Debenham B, Banerjee R, Fairchild A, Dundas G, Trotter T, Yee D. 2009 Canadian radiation oncology resident survey. Int J Radiat Oncol Biol Phys. 2012;82(4):1326–31.

28. Moran JM, Fraass BA. Introduction: Quality, Technology, and Outcomes in Radiation Oncology. Semin Radiat Oncol. 2012;22(1):1–2.

29. Semrau R, Hansemann K, Adam M, Andratschke N, Brunner T, Heinzelmann F, et al. Quality of training in radiation oncology in Germany: Results of a 2006 survey. Strahlentherapie und Onkol. 2008;184(5):239–44.

30. Dietzel CT, Jablonska K, Niyazi M, Gauer T, Ebert N, Ostheimer C, et al. Quality of training in radiation oncology in Germany: where do we stand? Strahlentherapie und Onkol. 2018;1–10.

31. Routsis D, Staffurth J, Beardmore C, Mackay R. Education and training for Intensity-modulated radiotherapy in the UK. Clin Oncol. 2010;22(8):675–80.

32. Coles CE, Spooner D. Lifelong Learning in Clinical Oncology Editorial Series: Introduction and Overview. Clin Oncol. 2011;23(5):309–11.

33. Rooney KP, McAleese J, Crockett C, Harney J, Eakin RL, Young VAL, et al. The impact of colleague peer review on the radiotherapy treatment planning process in the radical treatment of lung cancer. Clin Oncol (R Coll Radiol). 2015 Sep;27(9):514–8.

34. Casswell G, Shakir R, Macnair A, O’Leary B, Smith F, Rulach R, et al. UK Training in Clinical Oncology: The Trainees’ Viewpoint. Clin Oncol. 2018;30:602–4.

35. Lei M, Stokoe J, MacLeod N, Yates L, Mir R. Clinical oncology training: The trainees’ perspective. Clin Oncol. 2012;24(1):22–4.

36. Wei RL, Colbert LE, Jones J, Racsa M, Kane G, Lutz S, et al. Palliative care and palliative radiation therapy education in radiation oncology: A survey of US radiation oncology program directors. Pract Radiat Oncol. 2017;7(4):234–40.

37. Walls GM, McAleese J, Hanna GG. Referrals Patterns to an Oncology Clinical Advice Service. Clin Oncol. 2018;30(6).

38. Pieters RS. A Radiation Oncologist’s Story: High Tech Meets High Touch. J Palliat Med. 2014;17(6):737–8.

39. Compton J, Gaspar L, Shrieve D, Wilson L, Griem K, Amdur R, et al. Resident-reported brachytherapy experience in ACGME-accredited radiation oncology training programs. Brachytherapy. 2013;12(6):622–7.

40. Benstead K. What is Valuable for Specialist Registrars to Learn in Order to Become Good Consultant Clinical Oncologists? Clin Oncol. 2006;18(7):549–54.

41. Orio PF, Nguyen PL, Buzurovic I, Cail DW, Chen YW. Prostate Brachytherapy Case Volumes by Academic and Nonacademic Practices: Implications for Future Residency Training. Int J Radiat Oncol Biol Phys. 2016;96(3):624–8.

42. Leifer R, Bristow B, Puts M, Alibhai S, Cao X, Millar BA, et al. National Survey Among Radiation Oncology Residents Related to Their Needs in Geriatric Oncology. J Cancer Educ. 2017;1–5.

43. Matalon SA, Howard SA, Abrams MJ. Assessment of Radiology Training During Radiation Oncology Residency. J Cancer Educ. 2018;1–5.

44. Spraker MB, Nyflot M, Hendrickson K, Ford E, Kane G, Zeng J. A survey of residents’ experience with patient safety and quality improvement concepts in radiation oncology. Pract Radiat Oncol. 2017;7(4):e253–9.

45. Faivre JC, Bibault JE, Leroy T, Agopiantz M, Salleron J, Wack M, et al. Evaluation of the Theoretical Teaching of Postgraduate Radiation Oncology Medical Residents in France: a Cross-Sectional Study. J Cancer Educ. 2018;33(2):383–90.

46. Woodhouse KD, Volz E, Bellerive M, Bergendahl HW, Gabriel PE, Maity A, et al. The implementation and assessment of a quality and safety culture education program in a large radiation oncology department. Pract Radiat Oncol. 2016;6(4):e127–34.

47. Kagan AR, DeHaan ML. Director of Residency Training, Do Your Job! Am J Clin Oncol. 2011;34(3).

48. Bydder S, Bloomfield L, Dally M, Harris P, Dorset L, Semmens J. Preparing to sit the Royal Australia and New Zealand College of Radiologists Faculty of Radiation Oncology Fellowship Part 2 examination: The value of a workshop including practice and feedback. Australas Radiol. 2007;51(5):465–71.

49. Szumacher E, Warner E, Zhang L, Kane G, Ackerman I, Nyhof-Young J, et al. Ontario Radiation Oncology Residents’ Needs in the First Postgraduate Year-Residents’ Perspective Survey. Int J Radiat Oncol Biol Phys. 2007;69(2):512–7.

50. Pohar S, Fung CY, Hopkins S, Miller R, Azawi S, Arnone A, et al. American Society for Radiation Oncology (ASTRO) 2012 workforce study: The radiation oncologists’ and residents’ perspectives. Int J Radiat Oncol Biol Phys. 2013;87(5):1135–40.

51. Brower J V., Liauw SL, Reddy A V., Golden DW. Radiation oncology residency selection: A postgraduate evaluation of factor importance and survey of variables associated with job securement. Pract Radiat Oncol. 2017;7(6):425–32.

52. Tripuraneni P, Watson RL, Ang KK, Harrison L, Eifel P, Zietman A, et al. Intersociety Radiation Oncology Summit-SCOPE II. Int J Radiat Oncol Biol Phys. 2008;72(2):323–6.

53. Tam VC, Berry S, Hsu T, North S, Neville A, Chan K, et al. Oncology education in Canadian undergraduate and postgraduate medical programs: A survey of educators and learners. Curr Oncol. 2014;21(1):75–88.

54. IAEA. IAEA Syllabus for the Education and Training of Radiation Oncologists. Vienna; 2009.

55. Burmeister J, Chen Z, Chetty IJ, Dieterich S, Doemer A, Dominello MM, et al. The American society for radiation oncology’s 2015 core physics curriculum for radiation oncology residents. Int J Radiat Oncol Biol Phys. 2016;95(4):1298–303.

56. Xiao Y, De Amorim Bernstein K, Chetty IJ, Eifel P, Hughes L, Klein EE, et al. The American Society for Radiation Oncology’s 2010 Core Physics Curriculum for Radiation Oncology Residents. Int J Radiat Oncol. 2011;81(4):1190–2.

57. Klein EE, Gerbi BJ, Price RA, Balter JM, Paliwal B, Hughes L, et al. ASTRO’s 2007 Core Physics Curriculum for Radiation Oncology Residents. Int J Radiat Oncol. 2007;68(5):1276–88.

58. Winey B, Shih HA, Sahoo N, Lee A, Vapiwala N, Both S. Core physics competencies for proton therapy training of radiation oncology and medical physics residents and fellows. Int J Radiat Oncol Biol Phys. 2014;88(4):971–2.

59. Giuliani ME, Gillan C, Milne RA, Uchino M, Millar BA, Catton P. Determining an imaging literacy curriculum for radiation oncologists: An international delphi study. Int J Radiat Oncol Biol Phys. 2014;88(4):961–6.

60. Gillan C, Yip KWT, Adleman J, Giuliani M. Competency in Quality: Defining the Scope and Nature of Quality Competencies for Radiation Oncology Residency Programs. J Med Imaging Radiat Sci. 2016;47(2):2.

61. Turner S, Eriksen JG, Trotter T, Verfaillie C, Benstead K, Giuliani M, et al. Establishing a Global Radiation Oncology Collaboration in Education (GRaCE): Objectives and priorities. Radiother Oncol. 2015;117(1):188–92.

62. The Royal College of Radiologists. Clinical Oncology UK workforce census report 2016. London; 2016.

63. The Royal College of Radiologists. Clinical Oncology - the future shape of the specialty. London; 2014.

64. Kantor G, Huguet F, Toledano A, Lafond C, Quero L, Servagi S, et al. Radiation oncology training in France: Demography, analysis of motivations of the young specialists, evaluation of the training | Dynamique et évaluation de la formation des internes en radiothérapie en France en 2005. Cancer/Radiotherapie. 2005;9(6–7):435–43.

65. Barrett J. Building for the Future. Clin Oncol. 2009;21(8):573–4.

66. Röttinger E, Barrett A, Leer JW. Guidelines for the infrastructure of training institutes and teaching departments for radiotherapy in Europe. Radiother Oncol. 2004;70(2):123–4.

67. Burt LM, Trifiletti DM, Nabavizadeh N, Katz LM, Morris ZS, Royce TJ. Supply and Demand for Radiation Oncology in the United States: A Resident Perspective. Int J Radiat Oncol Biol Phys. 2017;97(2):225–7.

68. Smith BD, Haffty BG, Wilson LD, Smith GL, Patel AN, Buchholz TA. The future of radiation oncology in the United States from 2010 to 2020: Will supply keep pace with demand? J Clin Oncol. 2010;28(35):5160–5.

69. Zaorsky NG, Siglin J, Den RB, Keith SW, Showalter TN, Dicker AP, et al. The responsibilities of a chief resident in radiation oncology: Results of a national survey. Int J Radiat Oncol Biol Phys. 2013;87(3):460–1.

70. The Royal College of Radiologists. ORF Ideal Trainee Timetable. London; 2016.

71. The Royal College of Radiologists. Guide to job planning in clinical oncology, Third edition. 2015;42.

72. McKenzie JT, Imwalle LE. Initial results of a survey of radiation oncology residency programs. Int J Radiat Oncol Biol Phys. 2013;86(3):405–6.

73. Nikapota A, Rogers S, Sevitt T, Simonds H, Smith S. Six months in physics - A useful training opportunity? Br J Radiol. 2007 Sep 1;80(957):766.

74. Campbell G, Wynne CJ, Baggarley S. Covering the CSF space: Does teaching make a difference? Australas Radiol. 1999;43(1):73–5.

75. Rosenstein BS, Held KD, Rockwell S, Williams JP, Zeman EM. American Society for Radiation Oncology (ASTRO) Survey of Radiation Biology Educators in U.S. and Canadian Radiation Oncology Residency Programs. Int J Radiat Oncol Biol Phys. 2009;75(3):896–905.

76. Das I, Moskvin V. Variability of Physics Education in Radiation Oncology Medical Residency Programs. Vol. 9, Journal of the American College of Radiology : JACR. 2012. 835-838.e1 p.

77. Gaudet M, Jaswal J, Keyes M. Current state of brachytherapy teaching in Canada: A national survey of radiation oncologists, residents, and fellows. Brachytherapy. 2015;14(2):197–201.

78. Balter JM, Haffty BG, Dunnick NR, Siegel EL. Imaging opportunities in radiation oncology. Int J Radiat Oncol Biol Phys. 2011;79(2):342–7.

79. Mansouri A, Witiw CD, Badhiwala JH, Nassiri F, McDonald PJ, Kulkarni A V., et al. National Perspectives on the Training of Neurosurgery Residents in Stereotactic Radiosurgery. Can J Neurol Sci. 2017;44(1):51–8.

80. Leer JW, Noordijk EM. Training in radiotherapy in The Netherlands. Int J Radiat Oncol Biol Phys. 1992;24(5):837–9.

81. Smith AR. Radiation physics instruction for residents. Int J Radiat Oncol Biol Phys. 1992;24(5):851–2.

82. Cummings BJ. Principles of radiation oncology training and manpower requirements in Canada. Int J Radiat Oncol Biol Phys. 1992;24(5):833–5.

83. Eriksen JG, Leech M, Benstead K, Verfaillie C. Perspectives on medical education in radiation oncology and the role of the ESTRO School. Clin Transl Radiat Oncol. 2016;1:15–8.

84. Eriksen JG. Postgraduate Education in Radiation Oncology in Low- and Middle-income Countries. Clin Oncol. 2017;29(2):129–34.

85. Chalubinska-Fendler J, Fendler W, Luniewska-Bury J, Mlynarski W, Spych M, Fijuth J. Tackling the turmoil of transformation: Radiation oncology in poland. Int J Radiat Oncol Biol Phys. 2014;90(3):480–6.

86. Rodin D, Aggarwal A, Lievens Y, Sullivan R. Balancing Equity and Advancement: The Role of Health Technology Assessment in Radiotherapy Resource Allocation. Clin Oncol. 2017;29(2):93–8.

87. Mattes MD, Golden DW, Mohindra P, Kharofa J. Results of the 2013 association of residents in radiation oncology career planning survey of practicing physicians in the United States. J Am Coll Radiol. 2014;11(8):817–23.

88. Tharmalingam H, Vinayan A, Anyamene N. UK Training in Clinical Oncology : Tasters , Coasters and the National Recruitment Crisis National ‘ Taster Course .’ Clin Oncol. 2018;1–3.

89. Leung J, Rioseco P. Burnout, stress and satisfaction among Australian and New Zealand radiation oncology trainees. J Med Imaging Radiat Oncol. 2017;61(1):146–55.

90. Ciammella P, De Bari B, Fiorentino A, Franco P, Cavuto S, Alongi F, et al. The “BUONGIORNO” Project: Burnout Syndrome Among Young Italian Radiation Oncologists. Cancer Invest. 2013 Oct 1;31(8):522–8.

91. Blanchard P, Truchot D, Albiges-Sauvin L, Dewas S, Pointreau Y, Rodrigues M, et al. Prevalence and causes of burnout amongst oncology residents: A comprehensive nationwide cross-sectional study. Eur J Cancer. 2010;46(15):2708–15.

92. Nowakowski J, Borowiec G, Zwierz I, Jagodzinski W, Tarkowski R. Stress In An Oncologist’s Life: Present But Not Insurmountable : (Students Should Not Be Discouraged Choosing Oncology as Their Future Specialisation). J Cancer Educ. 2017;32(1):198–205.

93. Shanafelt TD, Gradishar WJ, Kosty M, Satele D, Chew H, Horn L, et al. Burnout and career satisfaction among US oncologists. J Clin Oncol. 2014 Mar;32(7):678–86.

94. Aggarwal S, Kusano AS, Carter JN, Gable L, Thomas CR, Chang DT. Stress and burnout among residency program directors in United States radiation oncology programs. Int J Radiat Oncol Biol Phys. 2015;93(4):746–53.

95. Folkert MR, Desai NB. Prostate Brachytherapy Procedural Training: Incorporation of Related Procedures in Resident Training and Competency Assessment. Brachytherapy. 2017 May 1;16(3):S66–7.

96. Ahmed AAA, Ramey SJ, Dean MK, Yoo SK, Holliday E, Deville C, et al. Resident satisfaction with radiation oncology training. Adv Radiat Oncol. 2018;3(3):234–9.

97. Mohamad O, Doke K, Marcrom S, Chen AM, Royce TJ, Meyer JJ. A Fellow’s Fate: Employment Outcomes of Radiation Oncology Fellowship Graduates. Int J Radiat Oncol Biol Phys. 2018;in press.

98. Balboni TA, Chen MH, Harris JR, Recht A, Stevenson MA, D’Amico A V. Academic Career Selection and Retention in Radiation Oncology: The Joint Center for Radiation Therapy Experience. Int J Radiat Oncol Biol Phys. 2007;68(1):183–6.

99. Goranov BB, Drew Y, Graham J, Iqbal MS, Kagzi M, Mahtab N, et al. Academic Opportunities within Clinical Oncology Training. Clin Oncol. 2013;25(7):446.

100. Gillespie EF, Panjwani N, Golden DW, Gunther J, Chapman TR, Brower J V., et al. Multi-institutional Randomized Trial Testing the Utility of an Interactive Three-dimensional Contouring Atlas Among Radiation Oncology Residents. Int J Radiat Oncol Biol Phys. 2017;98(3):547–54.

101. Alfieri J, Portelance L, Souhami L, Steinert Y, McLeod P, Gallant F, et al. Development and impact evaluation of an e-learning radiation oncology module. Int J Radiat Oncol Biol Phys. 2012;82(3):e573–80.

102. Sura K, Lischalk JW, Leckie J, Welsh JS, Mundt AJ, Fernandez E. Webinar-Based Contouring Education for Residents. J Am Coll Radiol. 2017;14(8):1074-1079.e3.

103. Urbański B. The future of Radiation Oncology: Considerations of Young Medical Doctor. Reports Pract Oncol Radiother. 2012;17(5):288–93.

104. Jaswal J, D’Souza L, Johnson M, Tay K, Fung K, Nichols A, et al. Evaluating the impact of a Canadian national anatomy and radiology contouring boot camp for radiation oncology residents. Int J Radiat Oncol Biol Phys. 2015;91(4):701–7.

105. Chino J, Doyle S, Marks LB. The anatomy of radiation oncology residency training. Int J Radiat Oncol Biol Phys. 2014;88(1):3–4.

106. Zumwalt ACA, Marks L, Halperin EEC. Integrating gross anatomy into a clinical oncology curriculum: the oncoanatomy course at Duke University School of Medicine. Acad Med. 2007;82(5):469–74.

107. Cabrera AR, Lee WR, Madden R, Sims E, Hoang JK, White LE, et al. Incorporating gross anatomy education into radiation oncology residency: A 2-year curriculum with evaluation of resident satisfaction. J Am Coll Radiol. 2011;8(5):335–40.

108. Chino JP, Lee WR, Madden R, Sims EL, Kivell TL, Doyle SK, et al. Teaching the anatomy of oncology: Evaluating the impact of a dedicated oncoanatomy course. Int J Radiat Oncol Biol Phys. 2011;79(3):853–9.

109. Bekelman JE, Wolden S, Lee N. Head-and-Neck Target Delineation Among Radiation Oncology Residents After a Teaching Intervention: A Prospective, Blinded Pilot Study. Int J Radiat Oncol Biol Phys. 2009;73(2):416–23.

110. Dewas S, Bibault J-E, Blanchard P, Vautravers-Dewas C, Pointreau Y, Denis F, et al. Delineation in thoracic oncology: a prospective study of the effect of training on contour variability and dosimetric consequences. Radiat Oncol. 2011;6(1):118.

111. Szumacher E, Harnett N, Warner S, Kelly V, Danjoux C, Barker R, et al. Effectiveness of Educational Intervention on the Congruence of Prostate and Rectal Contouring as Compared With a Gold Standard in Three-Dimensional Radiotherapy for Prostate. Int J Radiat Oncol Biol Phys. 2010;76(2):379–85.

112. Walker G V., Awan M, Tao R, Koay EJ, Boehling NS, Grant JD, et al. Prospective randomized double-blind study of atlas-based organ-at-risk autosegmentation-assisted radiation planning in head and neck cancer. Radiother Oncol. 2014;112(3):321–5.

113. Eriksen JG, Salembier C, Rivera S, De Bari B, Berger D, Mantello G, et al. Four years with FALCON - An ESTRO educational project: Achievements and perspectives. Radiother Oncol. 2014;112(1):145–9.

114. Mattes MD. The Evolving Role of Regional Radiation Oncology Societies in Resident Education. J Cancer Educ. 2015;30(3):428–31.

115. Bibault JE, Fumagalli I, Diaz O, Faivre JC, Leroy T, Pichon B, et al. The French Society of Young Radiation Oncologists: History, goals and perspective. Reports Pract Oncol Radiother. 2012;17(5):255–8.

116. Ostheimer C, Ebert N. OC-0328: Giving the next generation of Radiation Oncologists a voice - experiences from the young DEGRO. Radiother Oncol. 2018;127:S174.

117. Filippi AR, Alongi F, Ciammella P, De Bari B, Franco P, Livi L. A strategy for young members within national radiation oncology societies: The Italian experience (AIRO Giovani group). Reports Pract Oncol Radiother. 2012;17(5):259–61.

118. The Royal College of Radiologists. The Oncology Registrars’ Forum [Internet]. Available from: https://www.rcr.ac.uk/oncology-registrars-forum-1

119. Jones C. Northern Oncology Trainees Collaborative for Healthcare Research (NOTCH). 2017.

120. Brown LC, Laack TA, Ma DJ, Olivier KR, Laack NN. Multidisciplinary medical simulation: A novel educational approach to preparing radiation oncology residents for oncologic emergent on-call treatments. Int J Radiat Oncol Biol Phys. 2014;90(3):705–6.

121. Thaker NG, Kudchadker RJ, Swanson DA, Albert JM, Mahmood U, Pugh TJ, et al. Establishing high-quality prostate brachytherapy using a phantom simulator training program. Int J Radiat Oncol Biol Phys. 2014;90(3):579–86.

122. Rooney MK, Zhu F, Gillespie EF, Gunther JR, McKillip RP, Lineberry M, et al. Simulation as More Than a Treatment Planning Tool: A Systematic Review of the Literature on Radiation Oncology Simulation-Based Medical Education. Int J Radiat Oncol. 2018;

123. Giuliani M, Gillan C, Wong O, Harnett N, Milne E, Moseley D, et al. Evaluation of high-fidelity simulation training in radiation oncology using an outcomes logic model. Radiat Oncol. 2014;9(1):1–7.

124. Hunter RD, Maciejewski B, Leer JW, Kinay M, Heeren G. Training logbook for radiotherapy. Radiother Oncol. 2004;70(2):117–21.

125. Benstead K, Gilson D, Hanna L, Radhakrishna G, McAleer J, Bloomfield D, et al. Training in Clinical Oncology: Results of the Royal College of Radiologists’ Survey of New Consultants. Clin Oncol. 2012;24(10):143–8.

126. Holliday EB, Swanick CW, McAvoy SA, Walker G V., Crane CH, Mahajan A, et al. Development of a Comprehensive Clinical Radiation Oncology Resident Didactic Curriculum. J Am Coll Radiol. 2016;13(12):1514-1516.e6.

127. Adleman J, Niglas M, Millar BA. Leadership Education in Radiation Oncology Residency Training. Int J Radiat Oncol. 2017;99(2):E120–1.

128. Turner S, Janssen A, Chan M, Morris L, Mackenzie P, Shaw T, et al. Can radiation oncologists learn to be better leaders? Outcomes of a pilot Foundations of Leadership in Radiation Oncology program for trainees delivered via personal electronic devices. J Med Imaging Radiat Oncol. 2018;1–7.

129. Barker CA, Mutter RW, Shapiro LQ, Zhang Z, Wolden SL, Yahalom J. Contrast media use in radiation oncology: A prospective, controlled educational intervention study with retrospective analysis of patient outcomes. J Am Coll Radiol. 2010;7(12):967–74.

130. Burmeister J, Joiner M, Crosby M, Dominello M. Crouching physics, hidden bio: Incorporating physics and radiobiology education into the context of clinical radiation oncology. In: Medical Physics. Wiley-Blackwell; 2018. p. e120–706.

131. Yoo SK, Bian SX, Lin E, Batth SS, Ng LW, Andrade J, et al. Development of a Radiation Oncology Resident Continuity Clinic to Improve Clinical Competency and Patient Compliance. Int J Radiat Oncol Biol Phys. 2018;100(3):551–5.

132. Shakespeare TP, Mukherjee RK, Lu JJ, Lee KM, Back MF. Evaluation of an audit with feedback continuing medical education program for radiation oncologists. J Cancer Educ. 2005;20(4):216–21.

133. The Royal College of Radiologists. Trainee induction pack for specialty training in clinical oncology. London; 2016.

134. Diavolitsis VM, Shirazi H, Boyle J, Hayes JP. Residents’ educational needs during transition into radiation oncology residency. J Am Coll Radiol. 2011;8(11):785–8.

135. Thippu Jayaprakash K, Wood K, Shaffer R. Improving Clinical Oncology Trainees’ Radiotherapy Induction. Clin Oncol. 2016;28(12):e220.

136. Oncology Registrars’ Forum. Trainee Induction Pack. London;

137. Rozati H, Shah SP, Shah N. Smartphone Applications for the Clinical Oncologist in UK Practice. J Cancer Educ. 2015;30(2):367–73.

138. Calero JJ, Oton LF, Oton CA. Apps for radiation oncology. A comprehensive review. Transl Oncol. 2017;10(1):108–14.

139. Bibault JE, Leroy T, Blanchard P, Biau J, Cervellera M, Diaz O, et al. Mobile technology and social media in the clinical practice of young radiation oncologists: Results of a comprehensive nationwide cross-sectional study. Int J Radiat Oncol Biol Phys. 2014;90(1):231–7.

140. Koo K, Di Prospero L, Barker R, Sinclair L, McGuffin M, Ng A, et al. Exploring attitudes of Canadian radiation oncologists, radiation therapists, physicists, and oncology nurses regarding interprofessional teaching and learning. J Cancer Educ. 2014 Jun;29(2):350–7.

141. Akthar AS, Hellekson CD, Ganai S, Hahn OM, Maggiore RJ, Cohen EE, et al. Interdisciplinary Oncology Education: a National Survey of Trainees and Program Directors in the United States. J Cancer Educ. 2018;33(3):622–6.

142. Quick A, Walker C, Martin D. Competency Evaluation for Gynecologic Brachytherapy for Radiation Oncology Residents. Brachytherapy. 2017;16(3, Supplement):S62.

143. Hallock A, Bauman G, Read N, D’Souza D, Perera F, Aivas I, et al. Assessment and improvement of radiation oncology trainee contouring ability utilizing consensus-based penalty metrics. J Med Imaging Radiat Oncol. 2012;56(6):679–88.

144. Pötter R, Eriksen JG, Beavis AW, Coffey M, Verfaillie C, Leer JW, et al. Competencies in radiation oncology: A new approach for education and training of professionals for Radiotherapy and Oncology in Europe. Radiother Oncol. 2012;103(1):1–4.

145. Bulte C, Betts A, Garner K, Durning S. Student teaching: views of student near-peer teachers and learners. Med Teach. 2007 Sep;29(6):583–90.

146. Rashid MS, Sobowale O, Gore D. A near-peer teaching program designed, developed and delivered exclusively by recent medical graduates for final year medical students sitting the final objective structured clinical examination (OSCE). BMC Med Educ. 2011;11(1).
